# Inter-individual differences in the gene content of human gut bacterial species

**DOI:** 10.1186/s13059-015-0646-9

**Published:** 2015-04-21

**Authors:** Ana Zhu, Shinichi Sunagawa, Daniel R Mende, Peer Bork

**Affiliations:** European Molecular Biology Laboratory, Meyerhofstr. 1, 69117 Heidelberg, Germany; Daniel K. Inouye Center for Microbial Oceanography: Research and Education, University of Hawaii, 1950 East-West Road, Honolulu, HI 96822 USA

## Abstract

**Background:**

Gene content differences in human gut microbes can lead to inter-individual phenotypic variations such as digestive capacity. It is unclear whether gene content variation is caused by differences in microbial species composition or by the presence of different strains of the same species; the extent of gene content variation in the latter is unknown. Unlike pan-genome studies of cultivable strains, the use of metagenomic data can provide an unbiased view of structural variation of gut bacterial strains by measuring them in their natural habitats, the gut of each individual in this case, representing native boundaries between gut bacterial populations. We analyzed publicly available metagenomic data from fecal samples to characterize inter-individual variation in gut bacterial species.

**Results:**

A comparison of 11 abundant gut bacterial species showed that the gene content of strains from the same species differed, on average, by 13% between individuals. This number is based on gene deletions only and represents a lower limit, yet the variation is already in a similar range as observed between completely sequenced strains of cultivable species. We show that accessory genes that differ considerably between individuals can encode important functions, such as polysaccharide utilization and capsular polysaccharide synthesis loci.

**Conclusion:**

Metagenomics can yield insights into gene content variation of strains in complex communities, which cannot be predicted by phylogenetic marker genes alone. The large degree of inter-individual variability in gene content implies that strain resolution must be considered in order to fully assess the functional potential of an individual's human gut microbiome.

**Electronic supplementary material:**

The online version of this article (doi:10.1186/s13059-015-0646-9) contains supplementary material, which is available to authorized users.

## Background

The human intestinal microbiome plays an essential role in human health and disease [1] and complements human metabolism in many aspects [2]. Almost 10 million microbial genes were recently identified in 1,267 fecal samples, of which about only 300,000 are shared between more than 50% of the individuals [3]. The implied large gene content variation might be a consequence of species variation between individuals, but could also mirror strain variation of the same species. The latter has not been systematically investigated or even estimated yet although it is well known that strains of a given prokaryotic species can greatly differ in gene content [4], which in turn can lead to major phenotypic changes not only for the species, but also for the individual harboring it. For example, the bacterial polysaccharide utilization loci (PULs) can explain individual differences in degrading different carbohydrate components that are found in seaweed, fruits, and vegetables (for example, porphyran and xyloglycan) [5,6].

Studying gene content variation usually requires the isolation and sequencing of bacterial strains [4,7]. In a set of completely sequenced genomes from a given species, core genes (present in all strains) and accessory genes (missing in at least one strain) can be determined; the sum of these genes represents the ‘pan-genome’ [4,7,8]. Pan-genome studies typically use isolates that originate from limited geographical areas (for example, a strain collection from a single hospital [9] or a single country [10]) or focus on either pathogenic or clinically relevant isolates (which are often enriched in virulence genes [11,12] and reduced in genome size [13,14]). Consequently, the strains to be studied are often pre-selected according to specific criteria, due to the time and costs associated with their isolation and sequencing (for example, only around 9% of the more than 800 *Staphylococcus epidermidis* isolates that are available were sequenced based on their morphology [9]). Taken together, reference genomes that are currently available in public databases are skewed, often towards cultivable, clinically relevant and closely related strains, which hampers an unbiased analysis of gene content variation across bacterial species [4,9,10,15-22]. In contrast, metagenomic sequencing of uncultured microbial communities is not affected by these biases since strains are accessed directly as they exist in their natural environment. Using published metagenomic datasets, Schloissnig *et al.* [23] demonstrated that it is possible to characterize the variation landscape of gut microbial strains in a large cohort of individuals based on single nucleotide polymorphism (SNP) and on structural variation (SV). The study established individuality of the SNP variations, which appear temporarily stable [23], suggesting long-term persistence of individual-specific strains. However, the extent of gene content differences between strains of the same species across individuals remains to be shown.

In order to establish a baseline for functional differences of the microbiota between individuals that cannot be explained by species composition, we here apply the concept of core and accessory genes to abundant human gut bacterial species in their natural habitat, by using published metagenomic data [24-26]. We developed a procedure that is robust against biases, such as sequencing errors and stochastic effects, due to the application of stringent filtering procedures. We characterize gene content variation of the same species in different individuals in their genomic context and study the functions of the respective accessory genes. Finally, we use the large variations of capsular polysaccharide synthesis (CPS) and polysaccharide utilization (PUL) loci to illustrate the potential functional impact of gene content variability across individuals.

## Results and Discussion

### Data selection for metagenomic gene content variation analysis

**Figure 1:**
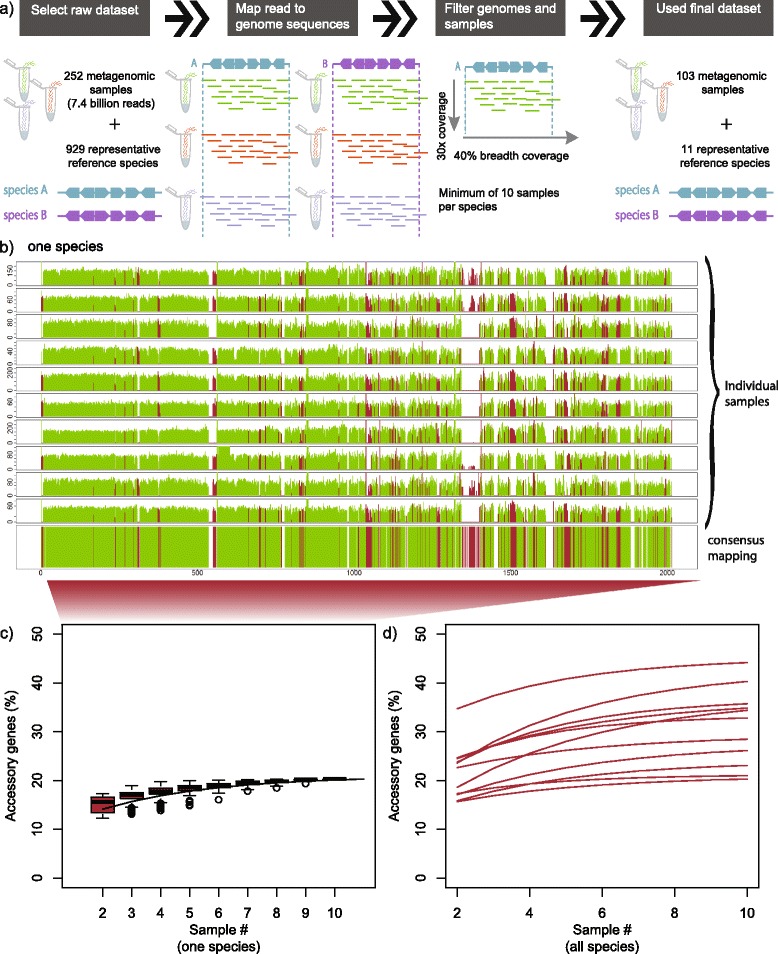
Summary of gene content determination pipeline. **(a)** Procedure of data selection for metagenomic gene content variation analysis. The initial dataset consisted of 252 metagenomic samples and a non-redundant set of reference genomes representative of 929 species based on 40 universal single copy marker genes. Metagenomic reads from each sample were aligned to each species and was followed by a multi-step filtering procedure used in sample and genome selection. The final dataset corresponded to 103 individuals that mapped to 11 species. **(b)** Diagram illustrating gene coverage of core and accessory genes of one species (*Dialister invisus*) for 10 individuals. The species is used to exemplify the typical variability in core and accessory genes coverage and location across the genome based on different individuals. Green denotes core genes, red denotes accessory genes, and white to missing genes. The bottom bar corresponds to the cross-samples consensus gene representing the core-accessory status, denoting the core, accessory and missing gene regions. **(c)** Boxplot shows the percentage of accessory genes (%) in *Dialister invisus* calculated from a subsampling procedure. The median values of different sample sizes were used to fit to the exponential regression model curve. **(d)** Shows the fitted exponential regression model for the 11 gut bacterial species and uses the same approach as in (c).

To enable the assessment of gene content variability between strains in unrelated individuals, we used 252 fecal metagenomes of 207 individuals from publicly available datasets (the NIH Human Microbiome Project [24], and the European Metagenomics of the Human Intestinal Tract consortium [25]). The metagenomics-based approach uses fragment recruitment to existing reference genomes, where a total of 7.4 billion reads from 252 samples were mapped to representative reference genomes from 929 species [23] (Figure 1a). Multiple filtering steps were applied to each sample to ensure high accuracy in species and gene assignment (see details in Material and Methods). Whenever more than one sample per individual was available, only one was chosen. Only 11 species from the phyla of Bacteroidetes and Firmicutes fulfilled our stringent filtering criteria and were sufficiently abundant in at least 10 individuals. In total, sufficient coverage of at least one of the 11 species was observed in 103 individuals. From the total pool of individuals where a species was sufficiently covered, 10 were randomly selected for each species and used throughout the study for comparability and to avoid potential sampling biases (Table 1 and Additional file 1).

**Table 1:** **Information about the metagenomic samples and reference genomes used in the current study**

**NCBI taxID**	**Representative genome strain name**	**Contigs (n)**	**Genes in reference (n)**	**Individuals (n)**	**Genes in metagenomes (n)**	**Gene mapped to OG (n)**	**OG (n)**
592028	*Dialister invisus DSM 15470*	1	2,015	16	1,905	1,343	1,081
657321	*Ruminococcus bromii L2-63*	1	1,852	22	1,807	1,324	1,031
511680	*Butyrivibrio crossotus DSM 2876*	31	2,576	13	2,493	1,768	1,253
657322	*Faecalibacterium prausnitzii SL3/3*	1	2,816	11	2,670	1,869	1,262
445970	*Alistipes putredinis DSM 17216*	11	2,795	58	2,790	1,649	1,266
537012	*Bacteroides cellulosilyticus DSM 14838*	66	5,771	15	5,542	4,125	2,247
483216	*Bacteroides eggerthii DSM 20697*	20	3,769	10	3,714	2,775	1,868
717959	*Alistipes shahii WAL 8301*	1	2,616	29	2,584	1,861	1,293
563193	*Parabacteroides sp. D13*	22	4,558	32	4,473	3,577	2,182
469586	*Bacteroides sp. 1_1_6*	71	5,648	41	5,639	4,232	2,468
537011	*Prevotella copri DSM 18205*	28	3,413	32	3,195	2,090	1,490

Description of the 11 representative reference genomes is summarized according to their NCBI TaxID and strain name. Statistics regarding the number of individuals, genes (both present in reference genome and seen in all metagenomes), contigs, orthologous groups, genes mapped to orthologous groups (OGs) are shown.

### High fraction of accessory genes that increases with genome size

For each of the 11 species, genes that were detected in all 10 individuals were categorized as ‘core’ and the remainder as ‘accessory’ (Table 2). We found that accessory genes are not evenly distributed across the genome (Figure 1b). Several regions show a high concentration of accessory genes and many are located in genomic islands (corresponding to 60 genomic islands up to 57 kb that were detected across the 11 species, Additional file 2). These are likely derived from mobile genetic elements, such as prophages, integrative plasmids, or integrative and conjugative elements [27].

**Table 2:** **Definitions used in the scope of the current study**

**Term**	**Definition**
Core gene	Species specific gene seen in all samples
Accessory gene	Species specific gene seen in some samples
Single-gene deletion block	Single gene missing in a sample when compared to the reference genome
Gene deletion block	Block of one or consecutive neighbour genes missing in a sample compared to reference genome
Large-gene deletion block	Deletion of 50 or more genes when compared to the reference genome in a sample
Consecutive-gene block	Consecutive genes that are present in a given sample
Individual	Refers to an individual gut sample for a given species (in Results and Discussion section)

Description of definitions used throughout the current study, the terminology was adapted from pan-genome studies to suit metagenomic studies.

**Figure 2:**
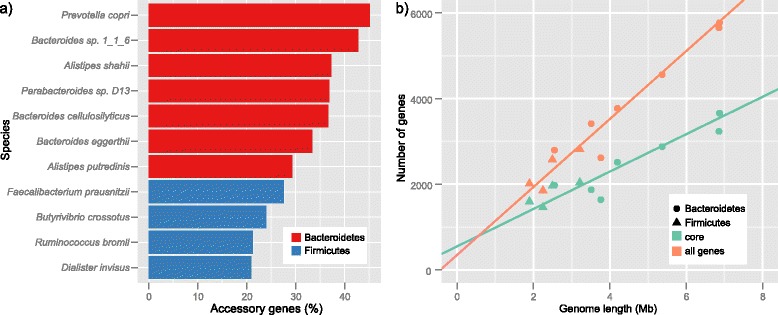
Percentage of accessory genes for 11 gut bacterial species. **(a)** The bars correspond to the percentage of accessory genes, which were calculated based on the asymptotic number originated from the exponential regression model. The values were estimated for the 11 gut bacterial species which are grouped according to their phyla. **(b)** Dot plot displays the relation between number of core genes or total number of genes and genome size. The graph shows that number of core genes also correlates with genome size; however the total number of genes grows faster with genome size than the number of core genes.

As accessory genes are known to vary greatly among bacterial species (reviewed in [4]), we first estimated the percentage of accessory genes in our set of 11 species in all 10 individuals. To this end, we used an approach consisting of a subsampling procedure, followed by exponential model fitting [21] in order to extrapolate the percentage of accessory genes (Figure 1c). The respective rarefaction curve tended to saturate or was close to saturation for all of the 11 species (Figure 1d), with the percentage of accessory genes in the range of 20.94% to 45.16% (average of 32.28%; Figure 2a). Note that these estimates are based on gene deletions compared to the respective reference genomes. Given that our approach does not take into account individual-specific genes that do not exist in the reference genome, these numbers should be considered as lower limits. These individual-specific genes can significantly increase the percentage of accessory genes, like in *Haemophilus influenza,* where strain-specific genes correspond to 19% of its gene repertoire [22]. Although it is impossible to estimate exact numbers of individual-specific genes, those that are specific to the reference genome and are not observed in any of the metagenomes, corresponding to an average of 3% in the species analyzed, can serve as minimum to be added to estimate gene content variability. Taken together, the high fraction of accessory genes per species as measured in their natural habitats is in a similar range as has been estimated from pan-genome studies [4,17,21,22,28]. In fact, it should be even higher as it is derived from a limited number of individuals and based on gene deletion analysis only, that is, gene insertions are not accounted for, as complex *de novo* metagenomic assembly of shotgun sequencing data cannot reconstruct complete genomes, which could be used to further advance our understanding of natural gut bacterial populations.

Regardless of the absolute numbers, Bacteroidetes were found to have a larger percentage of accessory genes compared to Firmicutes (*P* value <0.01). As Bacteroidetes have generally larger genomes, we separately compared the correlation of genome size with the number of core genes and all genes. The increase in the number of all genes was greater than the increase in the number of core genes (Figure 2b). This result is in line with a number of genome size ‘scaling laws’ that show gene functional classes scale differently with genome size [29-31], for example, that the number of transcription factors, two component system, signal transduction genes increase more than linearly with genome size [29]. It is also in agreement with the observation that larger genomes have higher rates of horizontal gene transfer compared to smaller genomes [32].

### Gene content variability in gut bacteria: metagenomics versus pan-genomics

**Figure 3:**
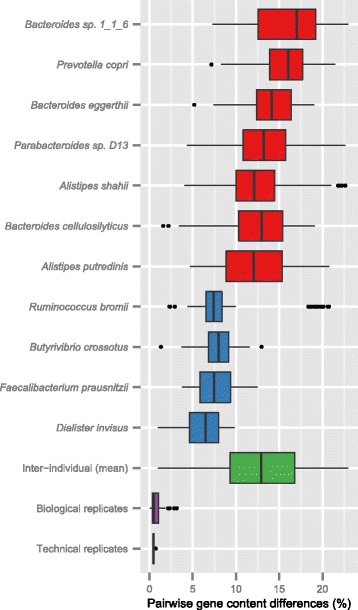
Inter-individual gene content variability of abundant bacterial species. For each species, the gene content differences between two individuals were calculated. Boxplots are colored according to phyla (red for Bacteroidetes and blue for Firmicutes), and species are sorted by decreasing mean. The inter-individual boxplot (green) shows pairwise comparisons of the same species between individuals. Biological replicates (purple) represent pairwise gene content comparisons of the same species in samples from the same individual at different time-points. Technical replicates represent gene content differences of *Prevotella copri* in four sequencing replicates of the same sample.

To quantify gene content differences of bacterial strains, we compared all pairs of individuals for each of the 11 species. When considering the set of genes that are present in either of the individuals and not in their intersection, the average gene content difference between individuals across all species was 13% ± 4.5% (mean ± SD) (Figure 3). This difference was considerably larger than the one observed between biological replicates (same individual, samples from different time points) and between technical replicates (same individual, same sample, different sequencing reactions), which were on average 0.81% and 0.51%, respectively, and statistically not significantly different (*P* value = 0.71, Figure 3).

Among the 11 species, *Bacteroides thetaiotaomicron* (represented by strain *Bacteroides* sp. 1_1_6 [23]) was found to have the highest (16%), and *Dialister invisus* (6%) the lowest average inter-individual variation. Again, these numbers represent lower limits due to the dependency on reference genomes for this estimate (see Material and Methods). Yet, for all 11 species, no two individuals share the same gene content, even when the analysis was extended to all 103 individuals.

To compare differences between strains in natural habitats with those derived from pan-genome studies, we collected genome sequences from any species belonging to the phyla of Firmicutes and Bacteroidetes (1,077 genomes assigned to pan-genomes of 35 species). We used this large dataset for comparison as for 10 out of the 11 species we investigated in our study, not enough genomes of other strains were available. We found a significantly higher gene content variation within our metagenomic dataset compared to published pan-genomes (a mean of 12.97% ± 4.51% and 10.69% ± 5.13%, respectively; *P* value <10^-16^, Additional file 3). The difference is even higher when pan-genomes from all available species (2,033 genomes assigned to 110 species pan-genomes) are computed (a mean of 9.19% ± 6.22% for completely sequenced genomes, *P* value <10^-16^, Additional file 3). Only for one species, *Parabacteroides* D. 13, genomes from eight different strains were available and differences between pairs of completely sequenced genomes and between pairs of metagenomes could be directly compared. The results indicate that pairwise differences of metagenomic samples (4.32% to 22.64%) are in similar ranges as those for completely sequenced genomes (6.68% to 20.61%), as shown in Additional file 4. Overall, these findings reveal no large systematic differences between our metagenomic estimations and the ones obtained from genomes of isolated strains.

The large gene content variation observed between gut strains implies considerable structural variation that need to be factored into the interpretation of metagenomic studies (note that gene content variation covers a large proportion of structural variation in prokaryotes due to high coding density). Furthermore, the structural variability of gut bacterial strains across individuals (using gene content variation as a proxy) is considerably larger compared to that of human genomes, as less than 1% of base pairs in structurally variable regions are different between two individuals [33]. This large gene content variation in gut strains could be due to a particularly high frequency of horizontal gene transfer (HGT) events in the gut compared to any other human body site or non-human habitats [34], which has been linked to antibiotic usage (for example, tetracycline) and inflammation [35,36]. Regardless of the underlying mechanisms, we found that the concept of individuality based on SNP variations [23] also holds at the level of gene content variations, at least within this limited dataset.

### Accessory genes are often derived from mobile elements and are enriched in functions associated with cell wall and cell membrane

In order to evaluate which functions are enriched in the pool of accessory genes, we assigned core and accessory genes to functional categories of clusters of orthologous groups (COGs) [37] (Additional file 5). As expected, accessory genes are enriched in functions that are often associated to mobile elements, such as recombination (this functional category includes several transposases and viral proteins), and defense mechanisms (for example, modification-restriction systems [38], ABC-type antimicrobial and multidrug transporters). Accessory genes were also enriched in functions associated to cell wall and cell membranes. As many as 33% of them encode glycosyltransferases, which are important for the modification of surface epitopes, such as capsular polysaccharides, O-antigens, and exopolysaccharides. The large panoply of glycosyltransferases may aid bacteria in the adaptation to colonize the gut environment [39,40]. In line with our observations, they have been already associated with HGT in gut-dwelling Bacteroidetes [39]. Lastly, accessory genes were enriched in genes with unknown function, and further exploration of their roles might reveal important phenotypes for which individuals differ.

### Highly abundant single gene deletions and their association to mobile elements

**Figure 4:**
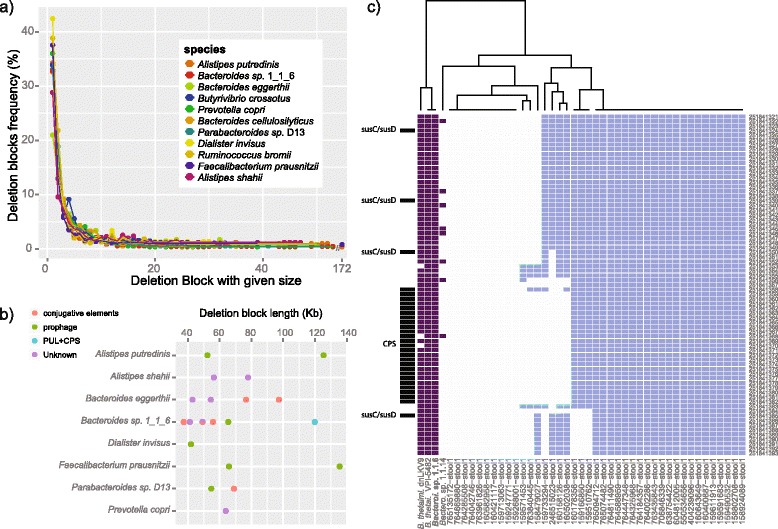
Gene deletion block size distribution. **(a)** Frequency of gene deletion blocks with a given number of contiguous absent genes (%) for 11 gut bacterial species. Each point corresponds to the mean frequency across 10 individuals for a given species. **(b)** Length of different deletion large deletion blocks across gut bacterial species. Several large deletion blocks are associated with prophage and conjugative elements and one containing PUL and CPS loci. **(c)** Heatmap showing an example of a large deletion block (containing 73 genes) in *Bacteroides thetaiotaomicron* species across the 41 individuals and four sequenced reference genomes. Metagenomic samples are represented in blue and reference genomes are represented in purple. The genes are represented by its NCBI sequence identifier (GI). The labeling of the reference genome used for metagenomic samples mapping is highlighted in bold. SusC/SusD and CPS annotations are based on those described in Xu *et al.* [46]. Upstream of the CPS there are three SusC/SusD genes, which can be associated with one or more PULs, and downstream of the CPS there is another PUL. These PULs are associated with plant carbohydrate degradation based on eggNOG and MetaCyc annotation (cf [68]). For CPS and both PULs related sub-regions, where the loci are present, they show a conserved modularity in all individuals except for one individual (where one SusC/SusD was missing). In agreement with metagenomic samples in the completely sequenced genomes, CPS and PULs loci are present in three genomes and absent in another one.

To gain mechanistic insights into the emergence of gene content variability we studied the architectural context of accessory genes based on gene deletion blocks [41,42]. We define a gene deletion block as a group of contiguous accessory genes that are absent in one individual when compared to the reference genome and a single-gene deletion block as a gene that is absent, but whose neighboring genes are certainly present in order to have a very strict criterion (Table 2). Such measurable deletion blocks can arise either by gene deletion in an individual or by gene insertion(s) in the reference genome. The number of gene deletion blocks and the number of genes they contained were determined in each metagenomic sample (Figure 4a and Additional file 6).

We found that the most frequent gene deletion blocks were single-gene deletions, corresponding to a mean of 33.74% of all blocks and 25% of all deleted genes. In the 11 gut species analyzed, several ATPases, transcription and recombination related proteins, such as retron-type reverse transcriptase, transcriptional regulators and recombinases, were at the top of this category (Additional file 7). These functional categories clearly link single gene deletions to mobile elements and the functional nature of the genes involved supports hypotheses that previously integrated mobile elements underwent erosion through deletion of their mobilization and integration machinery [43], although we cannot exclude that some of these are gene insertions in the reference genome.

### Accessory genes with functionality that imply phenotypic differences of an individual

At the other end of the size spectrum of gene deletions, we also detected large-gene deletion blocks with 50 or more genes in several species, the longest of which contained 172 genes, with deleted segment sizes in the range of 37 to 135 kilobases (Figure 4a, b and Additional file 8). In this category we found a mean of 21% of all deleted genes, which contain considerable numbers of operons, likely integrated into active mobile elements. Indeed, we found large integrons containing a likely queuosine biosynthetic pathway, several peptidases (for example, involved in lysis of cell wall peptidoglycan), and a toxin-antitoxin system (Additional file 9) that may confer functional differences between strains.

As it is also likely that differences in large gene blocks have phenotypic consequences in the respective individuals, we studied them in more detail. In total we detected 21 such large deletion blocks in eight species, with each species harboring between one and seven of them. Not unexpectedly, we found that many of them are associated with prophages of both Bacteroidetes and Firmicutes or conjugative transposable elements for Bacteroidetes (see Additional file 8) implying a mechanism for the transfer of functionality.

One of the large-gene deletion blocks found in *Bacteroides thetaiotaomicron* contains four susC/susD genes that are likely associated with two polysaccharide utilization loci (PULs) and a capsular polysaccharide synthesis locus (CPS) with 25 genes (Figure 4c). Bacterial PULs are important for foraging glycans and polysaccharides in the human intestine [44,45]. The two PULs we detected have been associated with plant carbohydrate degradation in *B. thetaiotaomicron* [46,47]. CPS loci are sensitive to the nutrient availability and are involved in the defense of the bacteria against environmental factors, such as the host immune system, phage attack and anti-peptide produced by the host or other bacteria [48,49]. Cross-individual comparison of the gene deletion patterns show that this large deletion block is further separated into at least three different sub-regions of consecutive-gene blocks (Figure 4c), with one corresponding to the CPS and two sub-regions containing PULs. We observed that each sub-region is present in some and missing in other individuals independent of the other sub-regions. This pattern is observed not only for the subset of 10 individuals that was comparatively analyzed, but extends to all individuals where *Bacteroides thetaiotaomicron* was detected according to our strict criteria (41 in total).

We further tested for the presence of these loci in three other completely sequenced genomes of this species (Figure 4c) and found that for *Bacteroides thetaiotaomicron* type strain VPI-5482 (ATCC 29148) the whole region is present, whereas some genes were missing in the other two sequenced strains. Thus, the variation in the gene content of this large deletion block in completely sequenced genomes of this species is in agreement with the variation observed between individuals. Before concluding about functional differences, paralogs have to be taken into account as they could compensate functionality at alternative loci. *B. thetaiotaomicron* produces more than 200 glycan-modifying enzymes, and several of them are paralogs [39], but we could not identify any paralogs for any of the genes within the large-gene deletion block. Thus, unless the individuals have inserts with corresponding functionality that we cannot measure, in some individuals CPS and the two PULs appear to be completely absent in *B. thetaiotaomicron,* limiting its potential for polysaccharide utilization and capsular polysaccharide synthesis.

More specifically, our findings regarding PULs support the idea that carbohydrate degradation is strain-specific as has also been found experimentally in Bifidobacteria [50], in which strains with different key enzymes are able to utilize different carbohydrate sources; these differences in carbohydrate utilization potential likely reflect differential niche adaptation [51-53]. For example, a study that compared *B. thetaiotaomicron* and *B. ovatus* showed that each species acquires niche-specific PULs that degrade different carbohydrate sources [51,53], which could be an effect of an individual’s dietary habits. The latter could also be influenced by strain-specific CPS architectures, as the expression of *B. thetaiotaomicron* CPSs has been coupled to diet changes [47,49,54] and as there is evidence of coordinated regulation of CPS with PULs [49]. The response of CPSs to dietary change is likely to help the bacteria to create a capsule with a similar glycan composition as the glycan landscape of individual’s gut, and affect the interaction between the bacteria and the immune system [48].

## Conclusion

This study addresses a current need to characterize gene content of species in their natural habitats, where bacterial species undergo natural selection and are influenced by a complex web of environmental factors. We have developed a method for gene deletion detection in metagenomic data and have demonstrated its applicability to complex microbial communities such as those inhabiting the human gut. Despite limited sample sizes, we show that in their natural habitats, strains of the same human gut bacterial species vary considerably in their gene content. The observed scaling of accessory genes with genome size (cf [29]) shows that the metagenomics-derived results are robust, also at the level of individual species. Given the considerable structural variation, it is likely that gene content might be as individual as SNP patterns have been proposed to be [23], although this concept needs to be validated in larger cohorts and with more species analyzed.

The fact that gene content variability of the same species implies potentially important functional differences that cannot be predicted by phylogenetic marker molecules alone [55] underlines the importance of global approaches like metagenomics in providing a complete functional fingerprint of an individual. The methods developed here can be applied to other environments in which the functional potential might be even more reflected in structural variations of strains than in the human gut. For example, soil samples harbor even more diverse microbial communities with larger genome sizes and hence should have an even higher fraction of accessory genes; the analysis of gene content variation of species in various environments might help to understand the gene flow within, but also between species in their natural habitats.

## Material and methods

### Data availability

Detailed information pertaining to the accessibility of the 252 metagenomic samples and reference genomes used in this study can be found in Supplementary Tables 1 and 2 in Schloissnig *et al.* [23]. The metagenomes are deposited in NCBI under the accession number BioProject PRJEB2054 (MetaHIT), and PRJNA43017 (HMP) in Additional file 1.

### Representative reference genomes selection and read mapping

Representative reference genomes were selected according to Schloissnig *et al.* [23,56]. In summary, a set of 1,511 prokaryote genomes was downloaded from GenBank and the MetaHIT Consortium on 4 July 2010. These genomes were clustered into a non-redundant set of 929 clusters based on 95% average nucleotide identity (ANI) of 40 universal single copy marker genes between sets of two genomes [57,58]. For each cluster, the genome recruiting the highest number of reads was selected to be the representative reference of that species [23,59].

Illumina reads from 252 metagenomic samples were mapped to the 929 representative reference genomes, with an alignment identity cut-off of 95% using Mosaik, according to Schloissnig *et al.* [23] (a summary of the pipeline is illustrated in Figure 1a).

### Filtering of genomes and individuals

Three data filtering steps were applied for each species-individual combination: (1) at least 40% of the genome of the species was covered by at least one read from an individual (breadth coverage) [23], and (2) a set of 40 universal single copy marker genes [57,58] had to be present. Both criteria are used to guarantee that the species detected corresponds to the reference genome mapped and not a close relative species with a similar genome composition. (3) At least 30× average genome coverage depth (the *de facto* standard for high coverage [60]) was required to assure determination of gene presence is not affected by sequencing depth. Only species with a minimum of 10 individuals (in the range of 10 to 58) were selected to increase statistical power. A final set of 11 species was used in the current study and for each species a random set of 10 individuals were chosen.

### Determination of core and accessory genes

The categorization of a gene as a core or accessory gene is based on presence or absence of the gene in all 10 individuals for each species, which can be seen in Figure 1b. A gene is considered present if it is covered with reads in at least 40% of its gene length. We set this gene length coverage filter to ensure that the gene is not called present due to spuriously assigned reads or reads originating from an orthologue belonging to a close relative species. To determine a cutoff for gene length coverage filter we compared the gene content between pairs of biological replicates (time-series) using cutoffs in the range of 0% to 100% in intervals of 10% (Additional file 10). A cutoff of 40% gene length coverage filter resulted in the lowest variability between biological replicates (Additional file 10) and affected 3% of the genes (Additional file 11).

This categorization of the genes into core and accessory was not affected by either the abundance or genome coverage of the species within the samples (calculated according to Schloissnig *et al.* [23]), since the fraction of accessory genes did not correlate with either of the two variables (correlation with coverage has a R = 0.08, *P* value = 0.82, see Additional file 12a, and correlation with abundance has a R = 0.07, *P* value = 0.84, see Additional file 12b).

### Estimation of percentage of accessory genes

To estimate the percentage of accessory genes we applied a subsampling procedure followed by model building. The subsampling procedure used random subsample sets of the 10 individuals with a defined sample size. The sample size was in the range of 2 to 10 and for each sample size all combinations of random subset of individuals were used. For each subsample set the ‘subsample-based fraction’ was calculated, that is the percentage of genes that were missing in at least one of the samples. For each sample size the mean subsample-based fraction was calculated and used to build the model. Two main models have been used in pan-genome studies, exponential regression model [21] and power law regression model [7]. In addition to these two models, we also tested using a negative exponential model and a spline function.

To evaluate the models we required the determination of the ‘expected fraction’, that is the expected fractions of accessory genes observed when the sample size increased beyond 10 individuals, which were used to compare with the estimated values generated by the models. The ‘expected fractions’ were calculated by using the same subsampling procedure as before but applied to all the available individuals, instead of the 10 randomly chosen. Therefore, the sample size was in the range of two to the total number of individuals where the species was observed; as the number of combinations increases rapidly with sample size, we randomly selected up to 500 combinations from each sample size. Next we compared the curve extrapolated from the model with the tendency observed in the ‘expected fraction’. The curves for the exponential and power law regression model are shown in Additional file 13. The exponential model predicts the curve closest to the expected fraction; exponential model showed a mean deviation from the expected fraction of 8% and the power law regression model a mean deviation of 12% and the other two models performed even worse. The exponential model was therefore chosen and the asymptotic number obtained from the model was used to predict the percentage of accessory genes. The exponential regression models tended to underestimate, so the values estimated here will correspond to a lower bound of the percentage of accessory genes.

### Estimation of the number of different genes between pairs of metagenomic samples and pairs of reference genomes

Inter-individual gene content variability was calculated for each of the 11 species by pair-wise comparisons between all pairs of individuals *A* and *B* in the following manner. The gene content difference between two individuals of one species was calculated by dividing number of genes present in only one of the individuals by the total number of genes present in either or both of the individuals (symmetric difference). Note that all observed genes have to be present in the reference genome to which the reads were mapped as well.

Gene content differences between biological and technical replicates were calculated using samples from different time-points of the same individual and multiple sequencing reactions for a given sample, respectively. For 33 individuals, one of the 11 species was detected in multiple time-points and their samples were used as biological replicates. The sample MH0006 was sequenced in four different lanes, and *Prevotella copri* had sufficiently high base pair coverage in each lane to pass our filtering criteria. Therefore data from each lane were used as technical replicates of gene content variability in *Prevotella copri*. Pairwise comparisons of gene content between biological and technical replicates were done in a similar fashion as described for inter-individual comparisons.

The outlined calculation was also applied for completely sequenced genomes. A total of 110 species were used after filtering for species where at least 10 completely sequenced genomes were available. For metagenomics, the calculation is dependent on the reference genome chosen to map the sequencing reads of samples. In order to emulate this reference genome dependency when surveying the gene content differences between completely sequenced genomes, we randomly selected one genome for each species as a ‘reference’ genome. This ‘reference’ genome was used as a third genome for each pair-wise genome comparison and all genes present in only one of the two compared genomes and not in the second one but present in the ‘reference’ genome were counted as ‘unique’ genes. The number of ‘unique genes’ was divided by the number of genes that were observed in the ‘reference’ genome and either or both of the two genomes. Metagenomic comparisons were compared to completely sequenced genome using a Wilcoxon-Mann-Whitney test.

### Functional annotation and enrichment test

Genes in each species were mapped to orthologous groups by using Blastp [61] (bitscore >60). The Cluster of Orthologous Group (COG) and Non-supervised Orthologous Group (NOG) from the eggNOG v3.0 pipeline [62] were used as orthologous groups. On average 71% of the genes per species have been assigned to orthologous groups in eggNOG database. The orthologous groups were further categorized into the COG functional categories [37]. Fisher test was used to find enrichment in COG functional categories and multiple testing was adjusted with FDR. Genes were also annotated with KEGG v62 [63] and MEROPs [64] by using Blastp [61] (bitscore >60). Genomic islands of the 11 species were detected using IslandViewer’s methods IslandPath-DIMOB and SIGI-HMM using default options [65].

### Accessory gene deletion block determination

In order to determine gene deletion blocks, we compared each of our metagenomes of a given species with the representative reference genomes, and located the genes that were absent in each metagenome. Contiguous absent accessory genes were clustered and named gene deletion blocks. When an accessory gene is absent and its two upstream and downstream closest neighbor genes are present, it is defined as single-gene deletion block. For each species we determine gene deletion blocks for each of the 10 individuals independently. Number of genes and number of gene deletion blocks were counted for each block size. For seven species the reference genomes were not completely assembled, and were composed of several contigs (Table 1). In these cases, the gene deletion block could only be counted within the context of a given contig. Hence, there is the possibility that gene deletion block could be split into two. Single-gene deletion blocks that occur in the start or end of a contig are not counted in order to not inflate the number of single-gene deletion blocks.

### Paralog determination within and between reference genome

Paralog determination for both within and between reference genomes was based on Alonso-Saez *et al.* [66]. The 95% ANI of 40 universal single copy marker genes mentioned above was used to find sequenced genomes that belong to the *B. thetaiotaomicron* species*.* Four genomes were found; apart from *Bacteroides sp.* 1.1.6 (the genome used in our study), we used *B. thetaiotaomicron* VPI-5482 (ATCC, NCBI TaxID 226186), *B. thetaiotaomicron* dnLKV9 (NCBI TaxID 1235785) and *Bacteroides sp*. 1.1.14 (NCBI TaxID 469585). These genomes were used to build *Bacteroides thetaiotaomicron-*specific orthologous groups (NOG) based on the eggNOG pipeline [67]. The genes were assigned to *B. thetaiotaomicron* NOG using Blastp [61] with a bit score cutoff of 60 and a 95% identity. Furthermore, to make sure that genes found in the large-deletion block do not even have distant paralogs within *Bacteroides thetaiotaomicron*, a less stringent cutoff of 40% identity and 80% protein length was also used. Annotation from the *B. thetaiotaomicron* VPI-5482 available in the MetaCyc database [68] was also used to annotate the *B. thetaiotaomicron* NOG.

## Additional files

Additional file 1:
**List of 10 individuals randomly chosen for each species.** For each of the 11 species the representative reference genome NCBI Tax ID and strain name are given together with the name of the 10 randomly selected individuals that were used throughout the study. Additional file 2:
**List of Genomic islands found using IslandViewer.** For each of the 11 species the location and length of genomic islands on genomic contigs are listed. Additional file 3:
**Variability between (1) sequenced reference genomes, (2) Bacteroidetes and Firmicutes reference genomes, and (3) metagenomic samples.** Boxplots showing the difference in number of genes (%) between pairs of: (1) sequenced reference genomes across the 110 bacterial species, (2) subset of sequenced reference genomes restricted to 35 Bacteroidetes and Firmicutes species, and (3) metagenomic samples across the 11 gut bacterial species used in this study. Only species with at least 10 sequenced reference genomes are included. Each boxplot corresponds to a pooling of pairwise comparisons between two samples from all the available species. The differences observed in metagenomic samples were significantly higher than in completely sequenced genomes, even when considering reference genomes from the same phyla. Additional file 4:
**Variability of gene content in** ***Parabacteroides*** **D.13 estimated using pairs of sequenced reference genomes and pairs of metagenomic samples.** Boxplots show differences in the number of genes (%) of *Parabacteroides* D.13 between pairs of sequenced reference genomes and pairs of metagenomic samples. The differences observed in metagenomic samples were in a similar range as in completely sequenced genomes. Additional file 5:
**Difference plot of orthologous groups functional categories between gene number of core and accessory genes.** The heatmap shows the difference between the numbers of core genes and the number of accessory genes belonging to a certain functional category. Darker green corresponds to functional categories with higher number of accessory genes compared to core genes. Species and functional categories are clustered according to the mean difference between gene number of core and accessory genes. Additional file 6:
**Cumulative number of genes in deletion blocks of a given size.** The total number of absent genes (%) that are located in a deletion block with size smaller or equal to the given block size (*x* axis) is plotted for 11 gut bacterial species. Each data point corresponds to the mean across the 10 individuals. The block size is defined by the number of genes absent in a given metagenomic sample and the block sizes were binned in bins of sizes multiples of 10. Additional file 7:
**Table with OG associated with single-gene deletion blocks.** Describes the number of genes found in single-gene deletion blocks that were associated with a given OG based on eggNOG v.3 [62]. The number of occurrences corresponds to a pooling of all the paralogs and orthologs found across the 11 species. The OGs are ranked according to the number of occurrences. Additional file 8:
**Table with mobile elements annotation associated with the 21 large deletion block.** Each large deletion block is identified by the reference genome NCBI Tax ID, contig, the start and end nucleotide positions. Annotation of the large deletion blocks is based on eggNOG [62], KEGG [63], and MetaCyc [68]. The deletion block is annotated as phage or conjugative transposon when the whole machinery is present or defined as such in MetaCyc. In contrast, when only some genes but not the whole machinery is present the deletion block is annotated as phage or conjugative transposon proteins. NA signifies that none of the genes within the large deletion block were annotated with functions associated with mobile elements. Deletion sizes are expressed in kilobases. Many of the large deletion blocks are associated with prophages and conjugative transposons. Additional file 9:
**Table with examples of function encoded in large deletion blocks that are likely to differ between strains.** The genes are grouped by regions, note that each region contain more genes, however for most of the remaining genes their function is currently unknown, only the relevant genes involved in queuosine biosynthetic pathway (Folate metabolism), toxin-antitoxin system and peptidases are described. Each gene is described with the genome of origin (NCBI Tax ID), the gene NCBI sequence identifier (NCBI GI), and their annotation to eggNOG [62], KEGG [63], MEROPs [64], and MetaCyc [68]. NA means that no functional annotation was found for the specific NCBI GI in the given database. Additional file 10:
**Gene content variability of abundant bacterial species between biological replicates using different gene length coverage filters.** Each boxplot represents the gene content differences between pairs of metagenomic samples of biological replicates (time-series) after applying a given gene length coverage filter. The gene length coverage filter is the fraction of a gene length that is covered with reads. The filter ranged between 0% and 100% (in intervals of 10%). The figure shows that the average variability is minimized at 40% gene length coverage filter. Additional file 11:
**Figure with gene length coverage filter from the 11 species.** Dotplot shows the percentage of genes that are called absent in all species-individual pairs if the gene length coverage filter cutoff is set at a certain *x* value, represented in the *x* axis. The gene length coverage filter is the fraction of a gene length that is covered with reads. With the chosen gene length coverage filter of 40%, 3% of all the genes that had reads mapped are considered as a result of spurious read mapping or homology with closely relative species and are regarded as absent. The plot was generated by pooling all metagenomic samples of the 11 species. Additional file 12:
**Percentage of accessory genes is not dependent on genome abundance nor genome coverage.** Boxplot shows the (a) depth of genome coverage and (b) relative abundance of each species within an individual. Also shown is the species fraction of accessory genes observed across 10 individuals. Species are sorted by the fraction of accessory genes and boxplot are colored according to which phylum a species belongs. Additional file 13:
**Percentage of accessory genes curve based on subsampling for each of the 11 species.** Each graph corresponds to a comparison between the ‘expected fraction’ and the percentage of accessory genes estimated by the models (exponential regression and power law regression). Within each graph the boxplots show the ‘expected fractions’; blue curve represents the fitting of the exponential regression model and the red curve represents the fitting of the power law regression model. ‘Expected fractions’ were calculated based on a subsampling procedure applied to all individuals. Exponential regression model and power law regression model were fitted to the median values of ‘subsample-based fraction’ that were based on 10 randomly chosen individuals. For small sample sizes both the curve fit similarly to the ‘expected fractions’, as sample size increases exponential regression model curve tend to underestimate the values while the power law regression model tend to overestimate. For larger sample sizes the difference between the values in the boxplot and two curves is smaller for the exponential regression model than the power law regression model.

## Abbreviations

ANI: Average nucleotide identity
COG: Cluster of orthologous group
CPS: Capsular polysaccharide synthesis locus
HGT: Horizontal gene transfer
NCBI TaxID: NCBI taxonomy identifier
NOG: Non-supervised orthologous group
OG: Orthologous group
PUL: Polysaccharide utilization locus
SNP: Single nucleotide polymorphism
