# Supplementary figures and images for: Inter-individual differences in the gene content of human gut bacterial species
Source: Genome Biol. 2015 Apr 21;16(1):82. doi: 10.1186/s13059-015-0646-9 (PMC4428241; doi:10.1186/s13059-015-0646-9)

Pairwise differences (%)

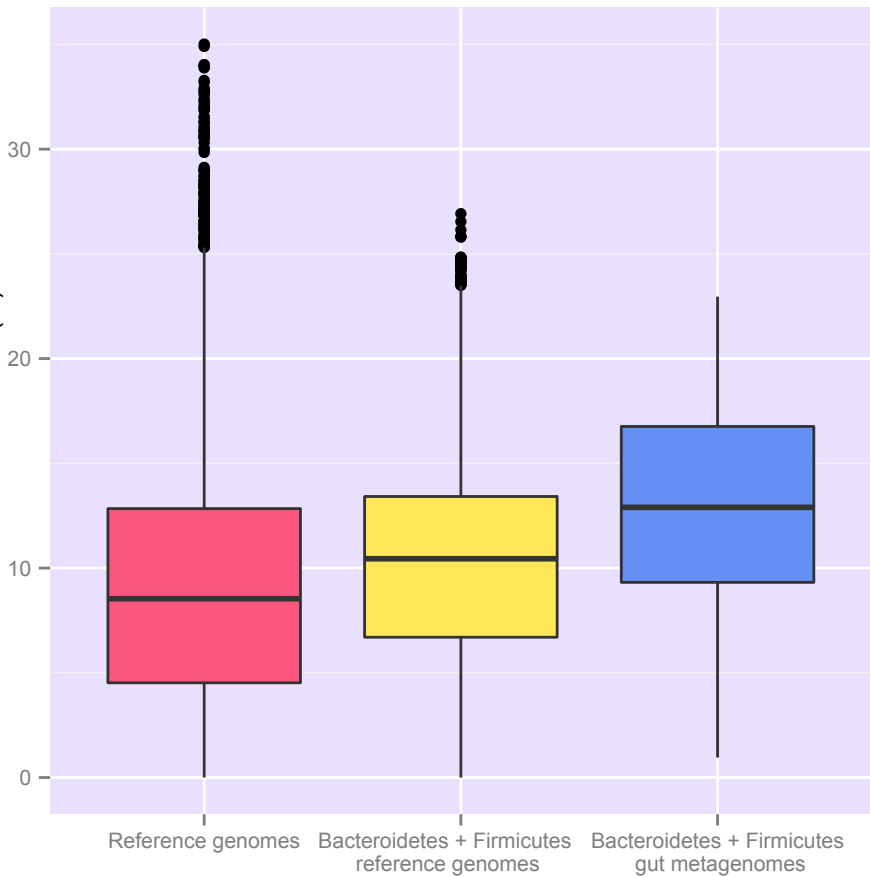

Supplement: Additional file 3: — Variability between (1) sequenced reference genomes, (2) Bacteroidetes and Firmicutes reference genomes, and (3) metagenomic samples. Boxplots showing the difference in number of genes (%) between pairs of: (1) sequenced reference genomes across the 110 bacterial species, (2) subset of sequenced reference genomes restricted to 35 Bacteroidetes and Firmicutes species, and (3) metagenomic samples across the 11 gut bacterial species used in this study. Only species with at least 10 sequenced reference genomes are included. Each boxplot corresponds to a pooling of pairwise comparisons between two samples from all the available species. The differences observed in metagenomic samples were significantly higher than in completely sequenced genomes, even when considering reference genomes from the same phyla. [file 13059_2015_646_MOESM3_ESM.pdf]

# Parabacteroides sp. D13

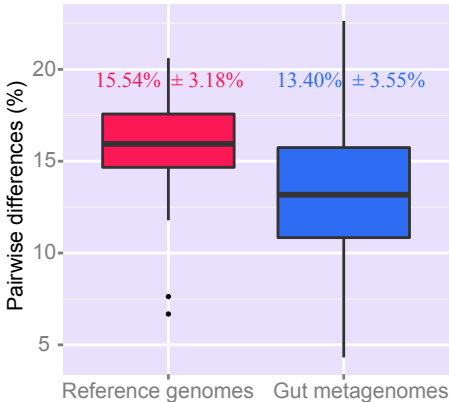

Supplement: Additional file 4: — Variability of gene content in Parabacteroides D.13 estimated using pairs of sequenced reference genomes and pairs of metagenomic samples. Boxplots show differences in the number of genes (%) of Parabacteroides D.13 between pairs of sequenced reference genomes and pairs of metagenomic samples. The differences observed in metagenomic samples were in a similar range as in completely sequenced genomes. [file 13059_2015_646_MOESM4_ESM.pdf]

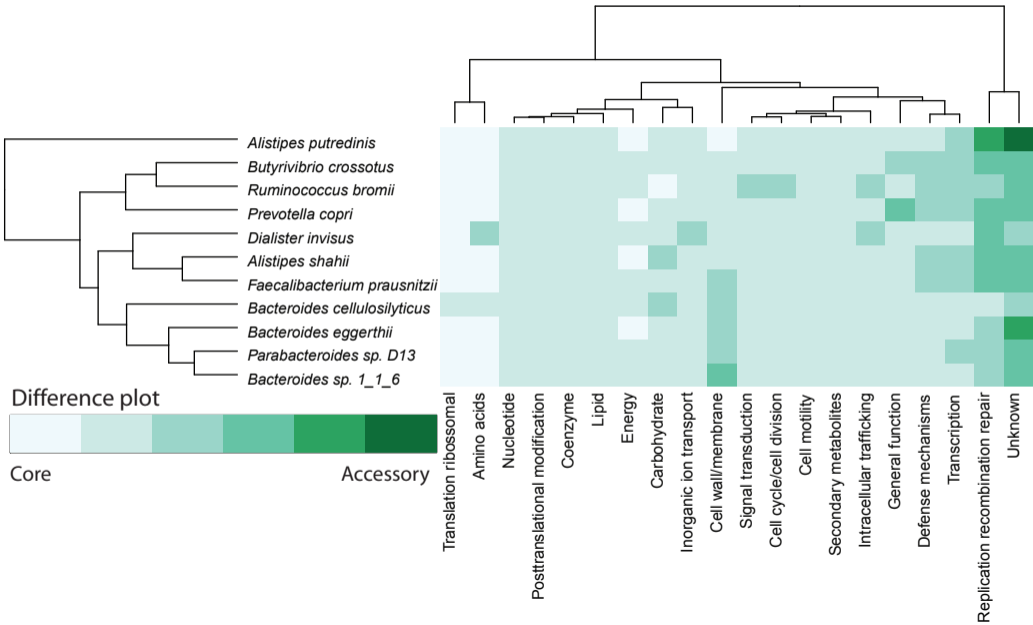

Supplement: Additional file 5: — Difference plot of orthologous groups functional categories between gene number of core and accessory genes. The heatmap shows the difference between the numbers of core genes and the number of accessory genes belonging to a certain functional category. Darker green corresponds to functional categories with higher number of accessory genes compared to core genes. Species and functional categories are clustered according to the mean difference between gene number of core and accessory genes. [file 13059_2015_646_MOESM5_ESM.pdf]

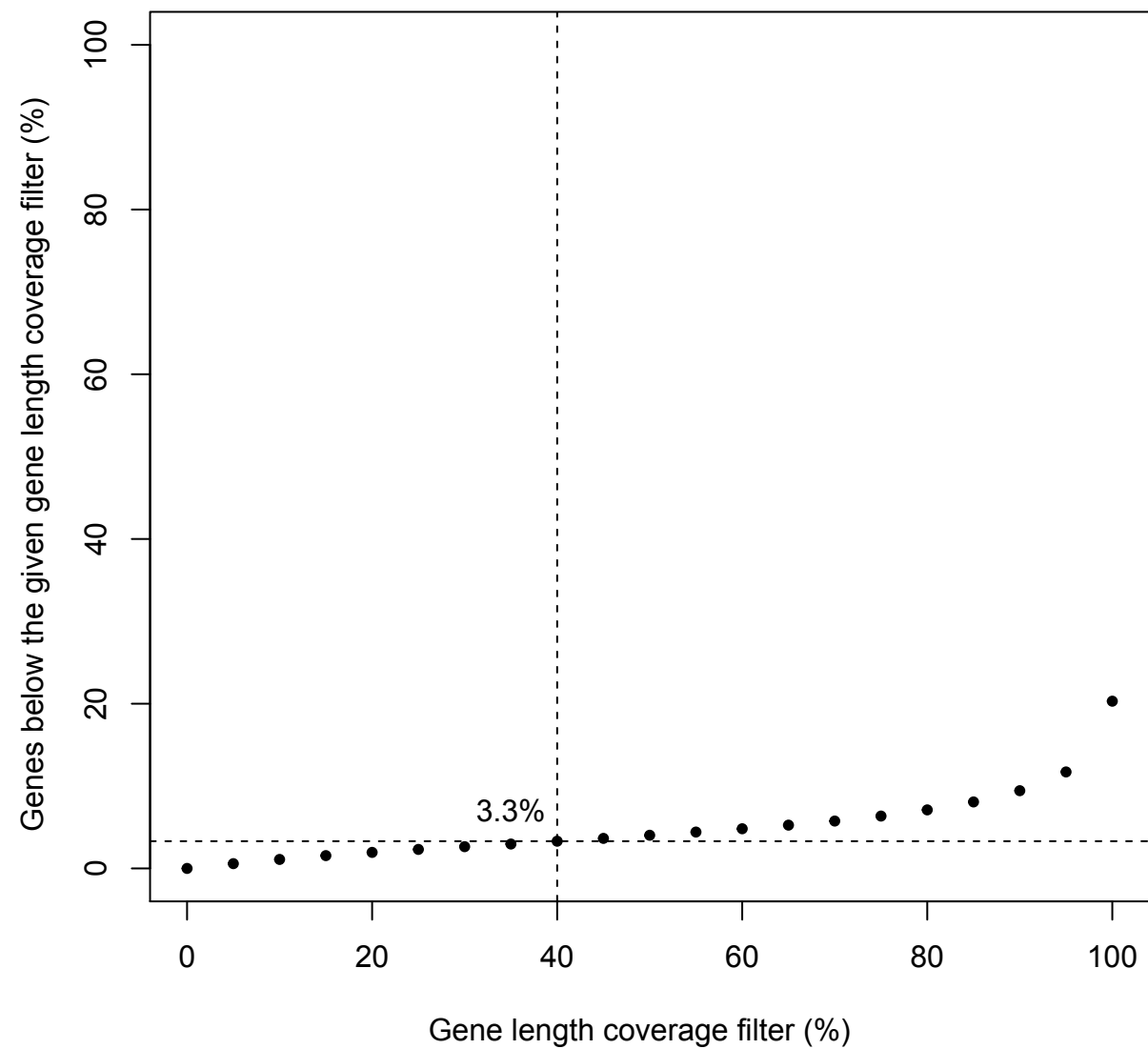

Supplement: Additional file 10: — Gene content variability of abundant bacterial species between biological replicates using different gene length coverage filters. Each boxplot represents the gene content differences between pairs of metagenomic samples of biological replicates (time-series) after applying a given gene length coverage filter. The gene length coverage filter is the fraction of a gene length that is covered with reads. The filter ranged between 0% and 100% (in intervals of 10%). The figure shows that the average variability is minimized at 40% gene length coverage filter. [file 13059_2015_646_MOESM10_ESM.pdf]

a)

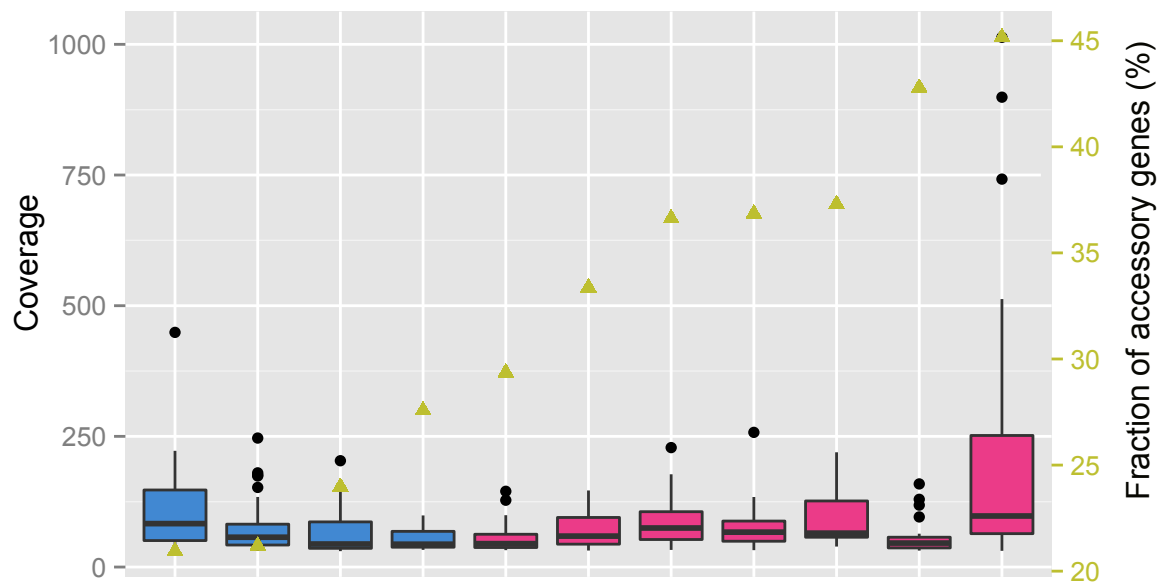

b)

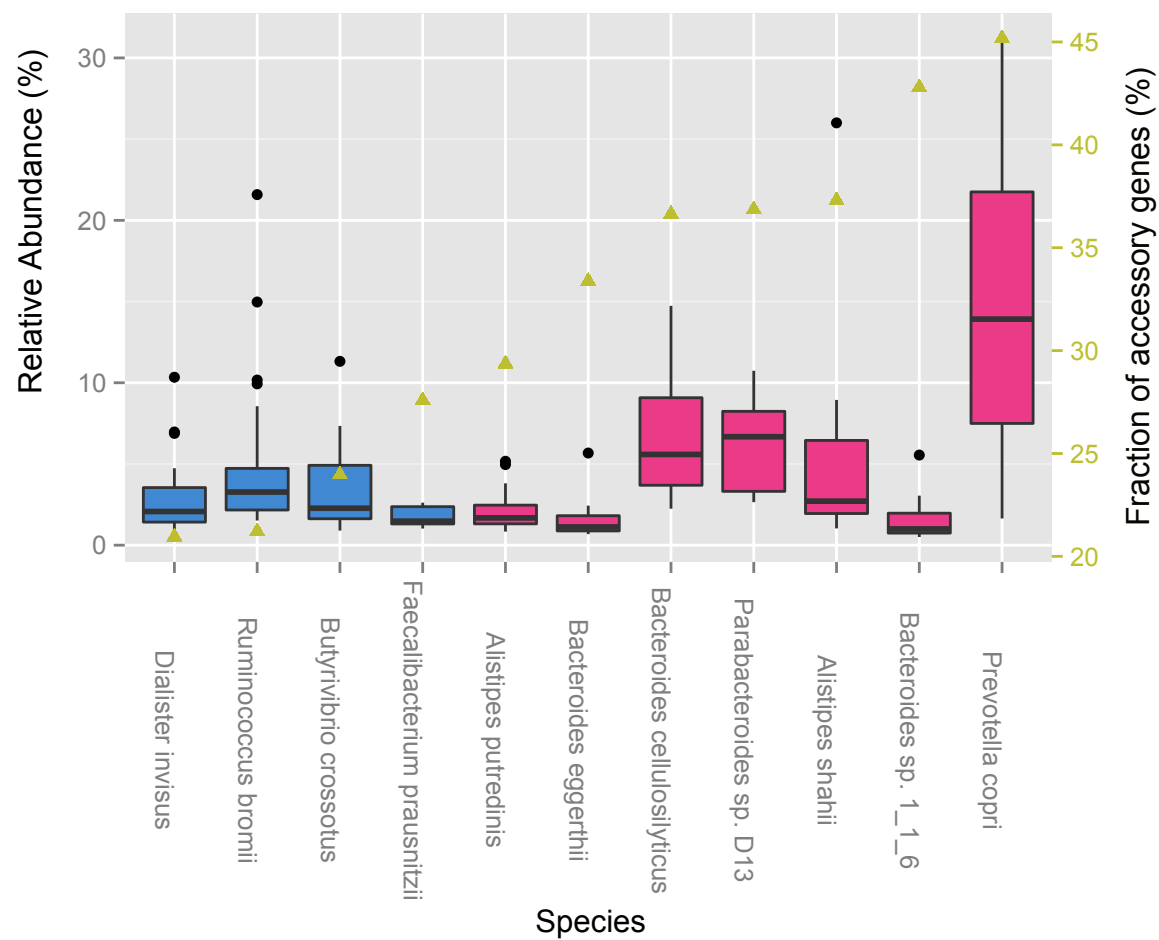

Phylum

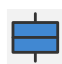

Firmicutes

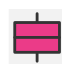

Bacteroidetes

Supplement: Additional file 12: — Percentage of accessory genes is not dependent on genome abundance nor genome coverage. Boxplot shows the (a) depth of genome coverage and (b) relative abundance of each species within an individual. Also shown is the species fraction of accessory genes observed across 10 individuals. Species are sorted by the fraction of accessory genes and boxplot are colored according to which phylum a species belongs. [file 13059_2015_646_MOESM12_ESM.pdf]
