# Supplementary material for: Inter-individual differences in the gene content of human gut bacterial species
Source: Genome Biol. 2015 Apr 21;16(1):82. doi: 10.1186/s13059-015-0646-9 (PMC4428241; doi:10.1186/s13059-015-0646-9)
Supplement: Additional file 6: — Cumulative number of genes in deletion blocks of a given size. The total number of absent genes (%) that are located in a deletion block with size smaller or equal to the given block size (x axis) is plotted for 11 gut bacterial species. Each data point corresponds to the mean across the 10 individuals. The block size is defined by the number of genes absent in a given metagenomic sample and the block sizes were binned in bins of sizes multiples of 10. [file 13059_2015_646_MOESM6_ESM.pdf]

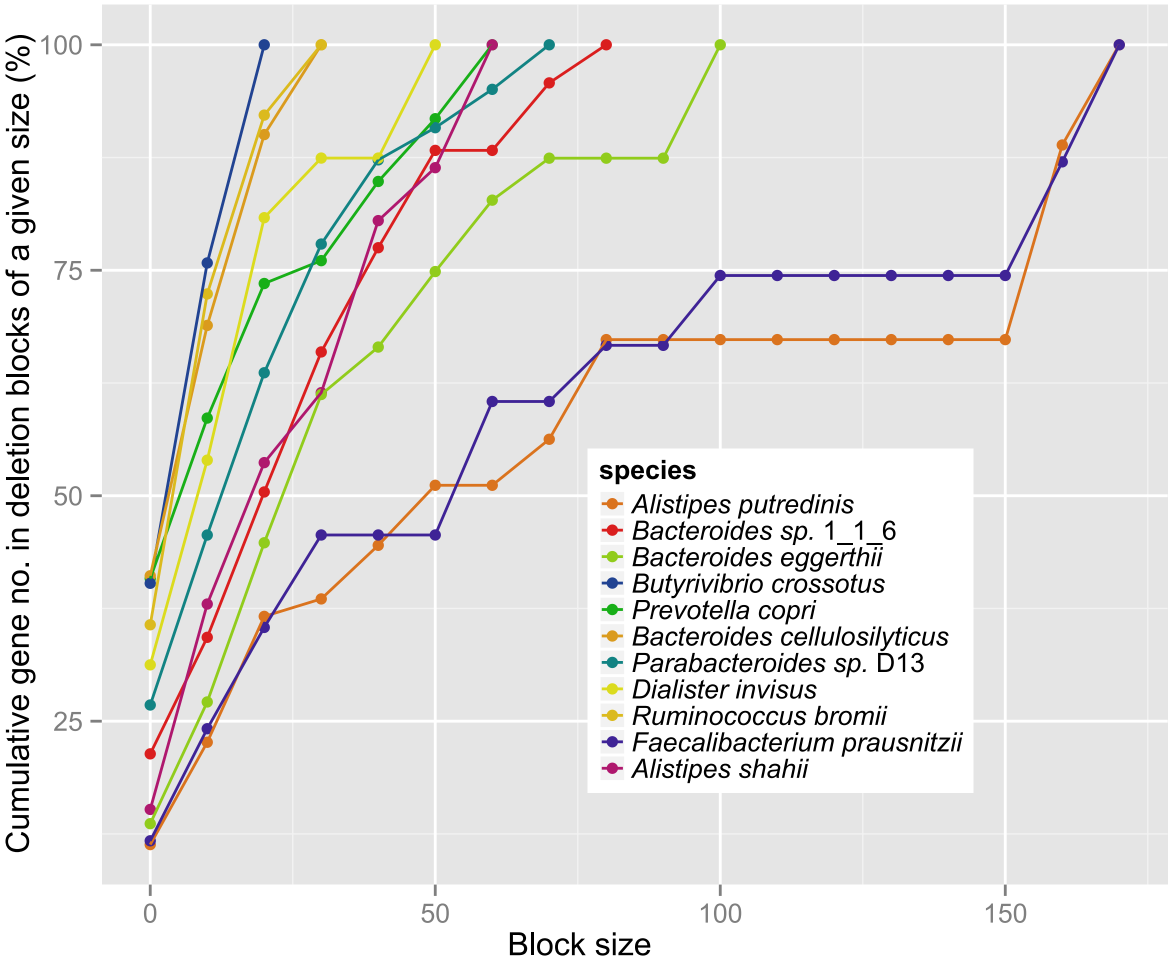
Cumulative gene no. in deletion blocks of a given size (%)
100
75
50
25
0
50
100
150
Block size
species
Alistipes putredinis
Bacteroides sp. 1_1_6
Bacteroides eggerthii
Butyrivibrio crossotus
Prevotella copri
Bacteroides cellulosilyticus
Parabacteroides sp. D13
Dialister invisus
Ruminococcus bromii
Faecalibacterium prausnitzii
Alistipes shahii
