# Supplementary material for: Inter-individual differences in the gene content of human gut bacterial species
Source: Genome Biol. 2015 Apr 21;16(1):82. doi: 10.1186/s13059-015-0646-9 (PMC4428241; doi:10.1186/s13059-015-0646-9)
Supplement: Additional file 13: — Percentage of accessory genes curve based on subsampling for each of the 11 species. Each graph corresponds to a comparison between the ‘expected fraction’ and the percentage of accessory genes estimated by the models (exponential regression and power law regression). Within each graph the boxplots show the ‘expected fractions’; blue curve represents the fitting of the exponential regression model and the red curve represents the fitting of the power law regression model. ‘Expected fractions’ were calculated based on a subsampling procedure applied to all individuals. Exponential regression model and power law regression model were fitted to the median values of ‘subsample-based fraction’ that were based on 10 randomly chosen individuals. For small sample sizes both the curve fit similarly to the ‘expected fractions’, as sample size increases exponential regression model curve tend to underestimate the values while the power law regression model tend to overestimate. For larger sample sizes the difference between the values in the boxplot and two curves is smaller for the exponential regression model than the power law regression model. [file 13059_2015_646_MOESM13_ESM.pdf]

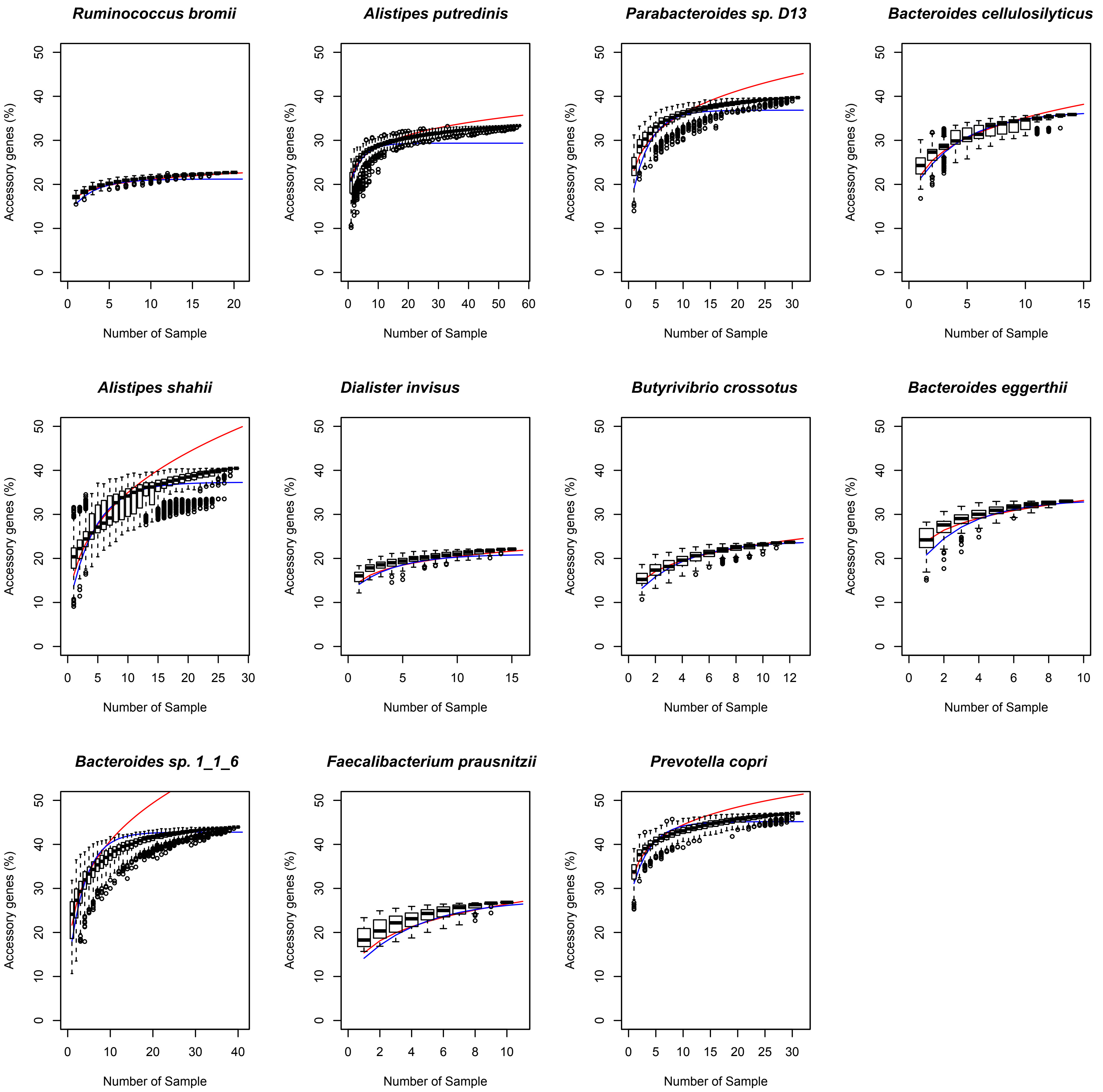

Ruminococcus bromii
Alistipes putredinis
Parabacteroides sp. D13
Bacteroides cellulosilyticus
Alistipes shahii
Dialister invisus
Butyrivibrio crossotus
Bacteroides eggerthii
Bacteroides sp. 1_1_6
Faecalibacterium prausnitzii
Prevotella copri
Accessory genes (%)
Number of Sample
